# 2-Deoxy-D-glucose inhibits lymphocytic choriomeningitis virus propagation by targeting glycoprotein N-glycosylation

**DOI:** 10.1186/s12985-023-02082-3

**Published:** 2023-05-31

**Authors:** Lucia Baďurová, Katarína Polčicová, Božena Omasta, Ingrid Ovečková, Eva Kocianová, Jana Tomášková

**Affiliations:** 1 Institute of Virology, Biomedical Research Center, Slovak Academy of Sciences, Bratislava, Slovakia; 2 Present Address: Functional Genomics and Proteomics of Plants, Central European Institute of Technology and National Centre for Biomolecular Research, Masaryk University, Brno, Czech Republic

**Keywords:** Arenavirus, LCMV, Cell metabolism, Glycolysis, 2-Deoxy-D-glucose, Glycoprotein, N-linked glycosylation, Virus-host interaction, Antiviral therapy

## Abstract

**Background:**

Increased glucose uptake and utilization via aerobic glycolysis are among the most prominent hallmarks of tumor cell metabolism. Accumulating evidence suggests that similar metabolic changes are also triggered in many virus-infected cells. Viral propagation, like highly proliferative tumor cells, increases the demand for energy and macromolecular synthesis, leading to high bioenergetic and biosynthetic requirements. Although significant progress has been made in understanding the metabolic changes induced by viruses, the interaction between host cell metabolism and arenavirus infection remains unclear. Our study sheds light on these processes during lymphocytic choriomeningitis virus (LCMV) infection, a model representative of the *Arenaviridae* family.

**Methods:**

The impact of LCMV on glucose metabolism in MRC-5 cells was studied using reverse transcription-quantitative PCR and biochemical assays. A focus-forming assay and western blot analysis were used to determine the effects of glucose deficiency and glycolysis inhibition on the production of infectious LCMV particles.

**Results:**

Despite changes in the expression of glucose transporters and glycolytic enzymes, LCMV infection did not result in increased glucose uptake or lactate excretion. Accordingly, depriving LCMV-infected cells of extracellular glucose or inhibiting lactate production had no impact on viral propagation. However, treatment with the commonly used glycolytic inhibitor 2-deoxy-D-glucose (2-DG) profoundly reduced the production of infectious LCMV particles. This effect of 2-DG was further shown to be the result of suppressed N-linked glycosylation of the viral glycoprotein.

**Conclusions:**

Although our results showed that the LCMV life cycle is not dependent on glucose supply or utilization, they did confirm the importance of N-glycosylation of LCMV GP-C. 2-DG potently reduces LCMV propagation not by disrupting glycolytic flux but by inhibiting N-linked protein glycosylation. These findings highlight the potential for developing new, targeted antiviral therapies that could be relevant to a wider range of arenaviruses.

**Supplementary Information:**

The online version contains supplementary material available at 10.1186/s12985-023-02082-3.

## Background

The obligate parasitic nature of viruses implies a dependence of their life cycles on the molecular and metabolic machinery of the host cells. After cell entry, viruses initiate the synthesis of their proteins, replication of the genome, and assembly of new viral progeny, placing high bioenergetic and biosynthetic demands on the host cell. To cope with this increased need for energy and macromolecular supply, viruses have developed numerous strategies for modulating cellular metabolic pathways, often similar to those observed in tumor cells.

In most cancer cells, glucose is predominantly metabolized via aerobic glycolysis (also known as the Warburg effect) to lactate instead of feeding the tricarboxylic acid (TCA) cycle [1]. Moreover, the Warburg effect-like changes are usually accompanied by an increased uptake of extracellular carbon (glucose and/or glutamine) and activation of anabolic pathways. As glucose carbon, diverted away from the TCA cycle, is mainly used for biosynthetic processes, glutamine may serve as an alternative carbon source anaplerotically refilling the TCA cycle and maintaining its function [2, 3]. Similarly to tumor cells, many viruses activate the process of aerobic glycolysis, divert glycolytic carbon to biosynthetic reactions, and stimulate glutamine utilization to replenish the TCA cycle intermediates. Some viruses have evolved strategies to use glutamine carbon to promote de novo fatty acid synthesis, amino acid supply, or glutathione production (reviewed in [4–7]). Thus, a unique metabolic signature is a characteristic feature of almost every virus. Interestingly, the pharmacological inhibition of various steps in central carbon metabolism has been shown as a promising strategy for suppressing the replication of different viruses (reviewed in [4, 6]).

Lymphocytic choriomeningitis virus (LCMV) is a prototypic representative of the *Arenaviridae* family, belonging to the genus *Mammarenavirus*. LCMV is an enveloped negative-strand RNA virus with a bi-segmented genome consisting of small (S-) and large (L-) segment. Both segments encode two open reading frames using an ambisense coding strategy, enabling regulated early and late gene expression determined by their orientation. Viral RNA-dependent RNA polymerase L and RING finger protein Z are encoded within the L-segment, while S-segment encodes nucleoprotein (NP) and glycoprotein precursor complex (GP-C), which is further proteolytically cleaved into stable signal peptide and viral envelope glycoprotein 1 and 2 (GP1 and GP2) (reviewed in [8, 9]). Even though LCMV infections are mostly asymptomatic and often go unnoticed, compelling evidence suggests that LCMV is a clinically significant human pathogen that can cause severe disease and consequences after prenatal infection [10, 11], as well as life-threatening conditions in immunocompromised individuals [12]. Moreover, several mammarenaviruses, such as Lassa virus or Junín virus, are known as the causative agents of human hemorrhagic fever outbreaks characterized by high mortality rates [9]. Since the currently available therapeutic options are mostly limited to supportive care and viral replication inhibitor ribavirin [13], there is an urgent need to design new strategies to prevent or cure arenavirus infections.

Given the relevance of virus-induced metabolic reprogramming of the host cells, it is surprising that we know very little about the role of cellular metabolism in LCMV pathogenesis. In this study, we investigated alterations in the glycolytic pathway following LCMV infection and elucidated metabolism-related requirements for LCMV propagation. We observed that LCMV infection increased the expression of enzymes involved in glucose utilization and uptake. However, depriving LCMV-infected cells of exogenous glucose or pharmacologically inhibiting glucose utilization had no effect on infectious virion production. Unexpectedly, we found that the routinely used glycolytic inhibitor 2-deoxy-D-glucose (2-DG) had a potent inhibitory effect on LCMV propagation. We demonstrate that the antiviral effect of 2-DG is predominantly caused by inhibition of viral glycoprotein N-linked glycosylation. Our results provide new insights into the complex interaction between LCMV and its host cells that may be useful in treating arenavirus infections.

## Materials and methods

### Cells and virus

Primary human lung fibroblasts (MRC-5) (ECACC 05072101), BHK21 cells (ATCC CCL-10), and Vero E6 cells (ATCC CRL-1586) were propagated and maintained in Dulbecco’s modified Eagle medium (DMEM) supplemented with 10% fetal calf serum (FCS) and 50 µg/ml gentamicin. Cells were grown in a humified atmosphere containing 5% CO_2_ at 37 °C. The Armstrong 53b strain of LCMV (obtained from the European Virus Archive) was propagated in BHK21 cells at MOI of 0.01, and titers were determined by focus forming assay (FFA) on Vero E6 cells. For the glucose depletion and inhibitor studies, DMEM lacking D-glucose, L-glutamine, sodium pyruvate, and phenol red (Gibco; A1443001) was used. The medium was supplemented with 2% dialyzed fetal bovine serum (dFCS) (HyClone), which is depleted of small molecules (including glucose), thus ensuring a negligible concentration of glucose present in the medium. For complete medium, 1 g/l D-glucose and 2 mM L-alanyl-glutamine were added.

### Reagents and antibodies

2-Deoxy-D-glucose (2-DG), sodium oxamate, D-mannose, and tunicamycin were purchased from Sigma and used at the indicated final concentrations. Peptide N-Glycosidase F (PNGase F) (P0708S) was obtained from New England BioLabs. Anti-β-actin antibody (#3700) was purchased from Cell Signaling Technology, anti-α-tubulin antibody (ab7291) was purchased from Abcam, anti-GP2 antibody (83.6) was a kind gift from Prof. Stefan Kunz (Institute of Microbiology of the University of Lausanne, Lausanne, Switzerland), anti-NP (M87) and anti-Z (MJ-3) are in-house made monoclonal antibodies [14]. Peroxidase-conjugated goat anti-mouse (A2554) was purchased from Sigma, and IRDye^®^ 680RD donkey anti-mouse (926-68072) was from Li-Cor.

### Virus infection

MRC-5 cells were plated at a density of 3.4 × 10^4^ cells per cm^2^. Twenty-four hours after seeding, the medium containing FCS was removed, cells were washed with phosphate-buffered saline without calcium and magnesium ions (PBS), and serum-free medium was added for 20–24 h prior to infection to synchronize the cell culture. Virus adsorption was carried out for 90 min at 37 °C in DMEM supplemented with 2% FCS at an MOI of 3, after which the infectious medium was removed, and the unbound viruses were removed by washing with PBS. Then the cells were cultivated in DMEM supplemented with 10% FCS. Mock-infected cells were treated under the same conditions as virus-infected cells. For virus infection growth kinetics, infectious media were harvested at various times post-infection (2–72 hpi) and used for measurement of LCMV titer by FFA. For the metabolic gene expression analysis, total cellular RNA was isolated at 4, 24, and 72 hpi.

### Nutrient starvation and inhibitor treatments

MRC-5 cells were LCMV-infected as described earlier. For the glucose starvation experiments, cells were washed with PBS and fed with a complete or glucose-free medium. For the inhibitor treatments, cells were washed and fed with complete medium or complete medium supplemented with either 2-DG (1, 3, 5, 10, and 20 mM), 2-DG (10 mM) + mannose (1 mM), mannose (1 mM), sodium oxamate (25 and 50 mM), or tunicamycin (0.5 and 5 µg/ml). The complete medium supplemented with an equivalent amount of DMSO was used as a control in the case of tunicamycin treatment. After 24-h cultivation, the infectious media were collected for LCMV titer quantification by FFA. For the virus infection time course study upon 2-DG treatment, cells were washed and then re-fed with a complete medium to which 10 mM 2-DG was added at various times post-infection (2–22 hpi). After 24-h incubation, the infectious media from all time points were harvested and used for viral titer quantification by FFA. Inhibition of viral infection was calculated as follows: % of inhibition = (1 − (inhibitor-treated cells)/(average of control cells)) × 100.

### Multi-step viral growth kinetics

MRC-5 cells were LCMV-infected as described earlier, however, at an MOI of 0.01. After 90 min, cells were washed with PBS and either complete medium or complete medium supplemented with 2-DG (1, 3, 5, 10, and 20 mM) was added. After 12, 24, 36 and 48-h post 2-DG addition, the infectious media were collected for LCMV titer quantification by FFA.

### Cell viability assay

A LIVE/DEAD™ Fixable Dead Cell Stain Kit (Invitrogen, L34969) was used to analyze the cell viability according to the manufacturer’s instructions.

### Reverse transcription-quantitative PCR (RT-qPCR)

Total RNA was isolated from mock- and LCMV-infected cells using the RNeasy Mini Kit (Qiagen), according to the manufacturer’s instructions, which also included the removal of genomic DNA. The quality and quantity of RNA were assessed using NanoDrop 2000 (Thermo Fisher Scientific). For cDNA synthesis, an equal amount of total RNA was reverse transcribed using the High-Capacity cDNA Reverse Transcription Kit (Thermo Fisher Scientific), with random hexamer primers. qPCR was performed on StepOnePlus™ Real-Time PCR System (Applied Biosystems) using SYBR Green PCR Master Mix (Applied Biosystems) and specific primers (Additional file 1: Table S1). The following reaction conditions were used: 95 °C 10 min; (95 °C 15 s, 60 °C 1 min) × 40 cycles. The RT-qPCR was performed in four biological replicates with three technical replicates for each sample. The comparative 2^−ΔΔCt^ method was used for data analysis, with β-actin (*ACTB*) serving as the reference gene, and mock-infected MRC-5 cells used as a calibrator.

Intracellular LCMV RNA was detected by amplification of the NP gene fragment within the S segment. To quantify viral RNA levels, a standard curve was created by amplifying known amounts of the NP gene fragment generated by RT-PCR from LCMV-infected MRC-5 cells. Seven consecutive dilutions, with a dilution factor 1:10, containing from 10^7^ to 10^1^ copies/reaction were prepared. Copy numbers of the LCMV genome in samples were determined by plotting Ct values onto the standard curve. All dilutions and individual samples were run in triplicate. The final result is expressed as copies per microliter of sample.

### Glucose uptake

The glucose uptake was measured by Glucose Uptake-Glo™ Assay (Promega) according to the manufacturer’s instruction. Briefly, cells were plated in 48-well plates (6 × 10^4^ cells/well) and were mock- or LCMV-infected as described earlier. At indicated times post-infection, cells were washed with PBS and treated with 1 mM 2-DG for 10 min. The uptake of 2-DG was terminated, and the whole-cell lysates were prepared, which were further used for intracellular 2-DG-6-P measurement according to the manufacturer’s protocol. Luminescence was recorded on the Synergy HT Reader (BioTek) using Gen5 software. Reactions were performed in four biological replicates.

### Lactate production

The lactate production was measured by Lactate-Glo™ Assay (Promega) according to the manufacturer’s instructions. Briefly, cells were plated in 96-well plates (2 × 10^4^ cells/well) and were mock- or LCMV-infected as described earlier. At indicated times post-infection, extracellular media were collected and further used for lactate measurement according to the manufacturer’s protocol. Luminescence was recorded on the Synergy HT Reader (BioTek) using Gen5 software. Reactions were performed in three biological replicates.

### Focus forming assay (FFA)

Vero E6 cells were seeded overnight into 12-well plates to achieve an 80% confluent monolayer the next day. Viral culture supernatants collected for LCMV titration were centrifuged to remove cell debris, tenfold serially diluted in DMEM supplemented with 2% FCS and added to Vero E6 cells. After 90 min viral adsorption period, the inoculum was aspirated, cells were washed with PBS and overlaid with carboxymethyl cellulose in DMEM supplemented with 5% FCS. Three days after infection, the cells were fixed in ice-cold methanol for 20 min at − 20 °C, washed with PBS, and blocked with 3% BSA in PBS for 1 h. After blocking, the cells were incubated for 1 h with an antibody against LCMV NP (M87). Subsequently, the cells were washed 3× with PBS and incubated for 45 min with peroxidase-conjugated goat anti-mouse antibody (dilution 1:10,000 in blocking buffer), then washed 3× with PBS and subjected to chemiluminescent detection using In-Vivo Imaging System FX (Kodak). LCMV titer was expressed as focus forming units per milliliter (FFU/ml).

### Virucidal assay

An infectious suspension of LCMV (1.3 × 10^5^ FFU/ml) was pre-incubated with 10 mM 2-DG in DMEM at 37 °C for 1 h. A parallel incubation of LCMV with DMEM alone served as a non-treated control. After the incubation period, infectious samples were diluted in DMEM (from 10^−1^ to 10^−6^), and each dilution was used to infect a monolayer of Vero E6 cells in a focus-forming assay.

### Western blot analysis

MRC-5 cells were LCMV-infected, and inhibitor treatments were performed as described earlier. At the time of analysis, cells were washed with cold PBS, harvested by a cell scraper, and resuspended in RIPA lysis buffer supplemented with protease and phosphatase inhibitors. The cell lysates were centrifuged at 14,000 rpm for 15 min at 4 °C, and supernatants were transferred to new tubes. Protein concentrations were determined using a Pierce™ BCA Protein Assay Kit (Thermo Fisher Scientific). Equal quantities of proteins (30 µg) were separated by SDS-PAGE (6–18% gradient gels) and then transferred to polyvinylidene difluoride membranes. Membranes were blocked with 5% nonfat dry milk in Tris-buffered saline with 0.1% Tween 20 (TBST) for 1 h and then probed with indicated primary antibodies diluted in Odyssey blocking buffer (Li-Cor) for 1 h at room temperature or overnight at 4 °C (dilutions: 1:500 for anti-GP2, 1:1,000 for anti-NP, 1:10,000 for anti-α-tubulin, 1:10,000 for anti-β-actin, and undiluted culture medium for anti-Z). Membranes were washed 5 × with TBST and then probed with peroxidase-conjugated goat anti-mouse or IRDye^®^ 680RD donkey anti-mouse secondary antibody diluted in Li-Cor blocking buffer (1:10,000) for 45 min at room temperature. Subsequently, the membranes were washed 5× with TBST, and immunoreactive proteins were visualized either by chemiluminescence or were scanned with an Odyssey CLx Imaging System (Li-Cor). The densitometric analysis of specific protein bands was performed using Image Studio™ Lite (Li-Cor).

### Peptide N-glycosidase F assay

Protein lysates from control or inhibitor-treated LCMV-infected cells were prepared as described earlier. Lysate aliquots (40 µg) were treated with PGNase F following the manufacturer’s instructions. Briefly, proteins were denatured with glycoprotein denaturing buffer at 100 °C for 10 min, then PNGaseF was added, and samples were incubated at 37 °C for 90 min. De-glycosylated proteins were further analyzed by western blotting.

### Statistical analysis

Data were subjected to statistical analysis using GraphPad Prism 9 software (https://www.graphpad.com/scientific-software/prism/). The differences between the two groups were determined using a two-tailed unpaired t-test or unpaired t-test with Welch’s correction. Analysis for three and more groups was performed using ordinary one-way ANOVA or Welch’s ANOVA (for groups with unequal variances), followed by an appropriate post hoc test (Tamhane’s, Sidak’s, or Tukey’s). Values of *P* ≤ 0.05 were considered significant. The statistical test used for a particular dataset is indicated in the figure legend.

For graphical representations of the statistical results, we utilized different methods depending on the number of groups being compared. The asterisk method was employed when comparing two groups, whereas the compact letter display method was used for comparisons involving more than two groups. In the compact letter display method, the same letter was assigned to all pairs of groups that were statistically insignificant. Conversely, if two groups do not share the same letter, they were considered to be significantly different.

## Results

### LCMV infection alters the expression of genes encoding glucose transporters and glycolytic enzymes

Glucose is one of the most important bioenergetic carbon sources in the cell, therefore, its uptake and metabolic utilization are modulated by many viruses (reviewed in [4, 6]). To investigate such alterations during LCMV infection, we analyzed virus-induced changes in the expression of several genes encoding proteins involved in glucose transport and in the process of glycolysis. We used primary human lung fibroblasts (MRC-5) for these experiments, as transformed or immortalized cells have significantly altered metabolism and thus are of limited value for studying virus-induced metabolic changes [3, 15]. Primary cells are, therefore, a more biologically relevant model for this purpose, as they better mimic the in vivo metabolic changes during viral infection. In addition, employing primary cell lines with unaffected metabolic profiles is particularly beneficial for exploring the mechanisms of viral pathogenesis, as well as for testing the efficacy of novel antiviral drugs that might target specific metabolic pathways.

**Fig. 1:**
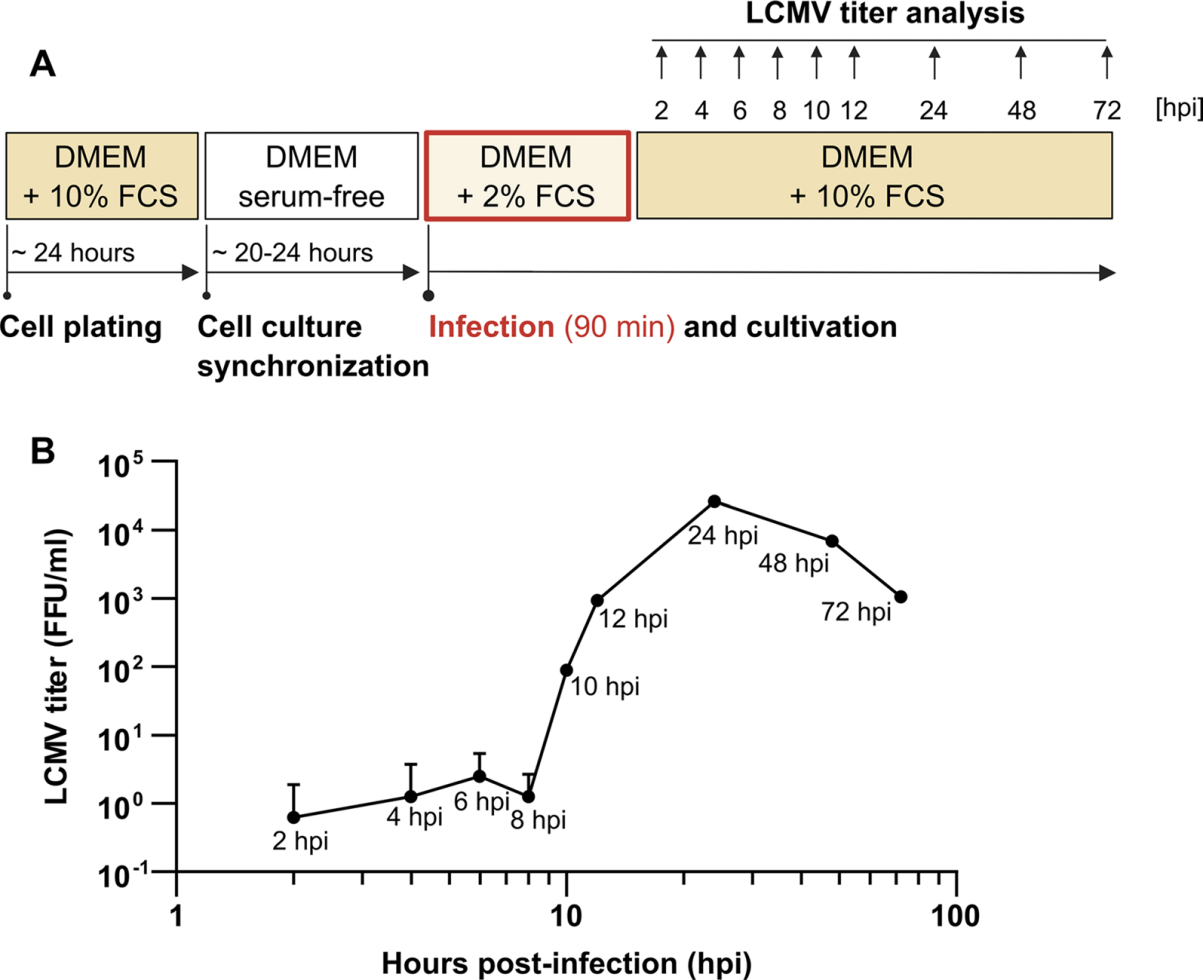
LCMV growth kinetics in MRC-5 cells. **A** Schematic of the experimental design. **B** MRC-5 cells were infected with LCMV at an MOI of 3. After adsorption, the inoculum was removed and replaced with fresh medium. Infected supernatants were collected at the indicated time points after infection, and LCMV titers were determined by a focus-forming assay. Data from two independent experiments are shown as a mean with SD. FFU/ml, focus-forming units per milliliter

Samples of mock- and LCMV-infected cells at a multiplicity of infection (MOI) of 3 (MOI was optimized to ensure near-complete infection of the cell population) were harvested at distinct time intervals for gene expression analysis. Based on the LCMV infection growth kinetics in MRC-5 cells (Fig. 1), we chose time intervals corresponding to infection stages when LCMV replication cycle has not been completed yet and no progeny virions are produced (4 h post-infection, hpi), when maximum virion production occurs (24 hpi), and when infectious virions production decreases (72 hpi).

The uptake of exogenous glucose into mammalian cells is mediated by the facilitative sugar transporters (GLUTs). Class I GLUTs (GLUT1-4) are the most well-characterized glucose transporters that differ in the spatiotemporal expression pattern and affinity towards glucose or other sugars (reviewed in [16]). GLUT1 mediates basal cellular glucose uptake, whereas GLUT3 is a high-affinity glucose transporter often found within tissues with high energy demand [17]. Interestingly, GLUT1 and GLUT3 were shown to be important in accelerating metabolic changes in both cancer and virus-infected cells [18–21]. To examine whether *GLUT1* and *GLUT3* expression is altered in LCMV-infected cells, total RNA was isolated, and relative transcript levels were determined using reverse transcription-quantitative PCR (RT-qPCR).

**Fig. 2:**
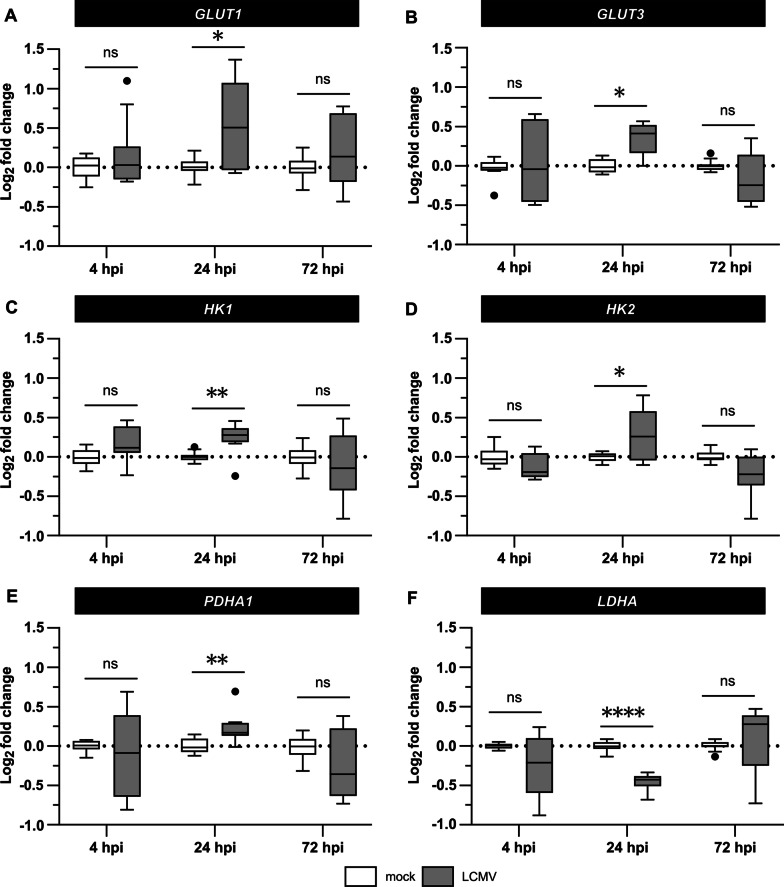
LCMV infection induces changes in the expression of genes involved in glucose uptake and utilization. MRC-5 cells were mock- or LCMV-infected at an MOI of 3 and the mRNA levels of *GLUT1* (**A**), *GLUT3* (**B**), *HK1* (**C**), *HK2* (**D**), *PDHA1* (**E**), and *LDHA* (**F**) were quantified by RT-qPCR at indicated times after infection. Gene expression levels normalized to β-actin (*ACTB*) were determined according to the 2^–∆∆Ct^ method. Data were log_2_ transformed and expression values from four independent experiments are shown as a box plot with Tukey’s whiskers. The median value and outliers are depicted by the horizontal line and solid dots, respectively. Statistical differences between groups were analyzed using Welch’s ANOVA with Tamhane’s T2 post hoc test. *P ≤ 0.05, **P ≤ 0.01, ***P ≤ 0.001, ****P ≤ 0.0001, *ns* not significant, *hpi* hours post-infection

We observed a modest but statistically significant increase in the *GLUT1* and *GLUT3* expression at 24 hpi (Fig. 2A, B). At the same time, the expression of hexokinase 1 and 2 (*HK1*, *HK2*) was also increased (Fig. 2C, D), implying that the imported glucose is phosphorylated and can be used in downstream cellular metabolic processes. The expression of E1α (*PDHA1*), a catalytic subunit of the pyruvate dehydrogenase complex, and lactate dehydrogenase A (*LDHA*) were up- and downregulated at 24 hpi, respectively (Fig. 2E, F), indicating a preferential flux of glucose into the TCA cycle instead of lactate production.

**Fig. 3:**
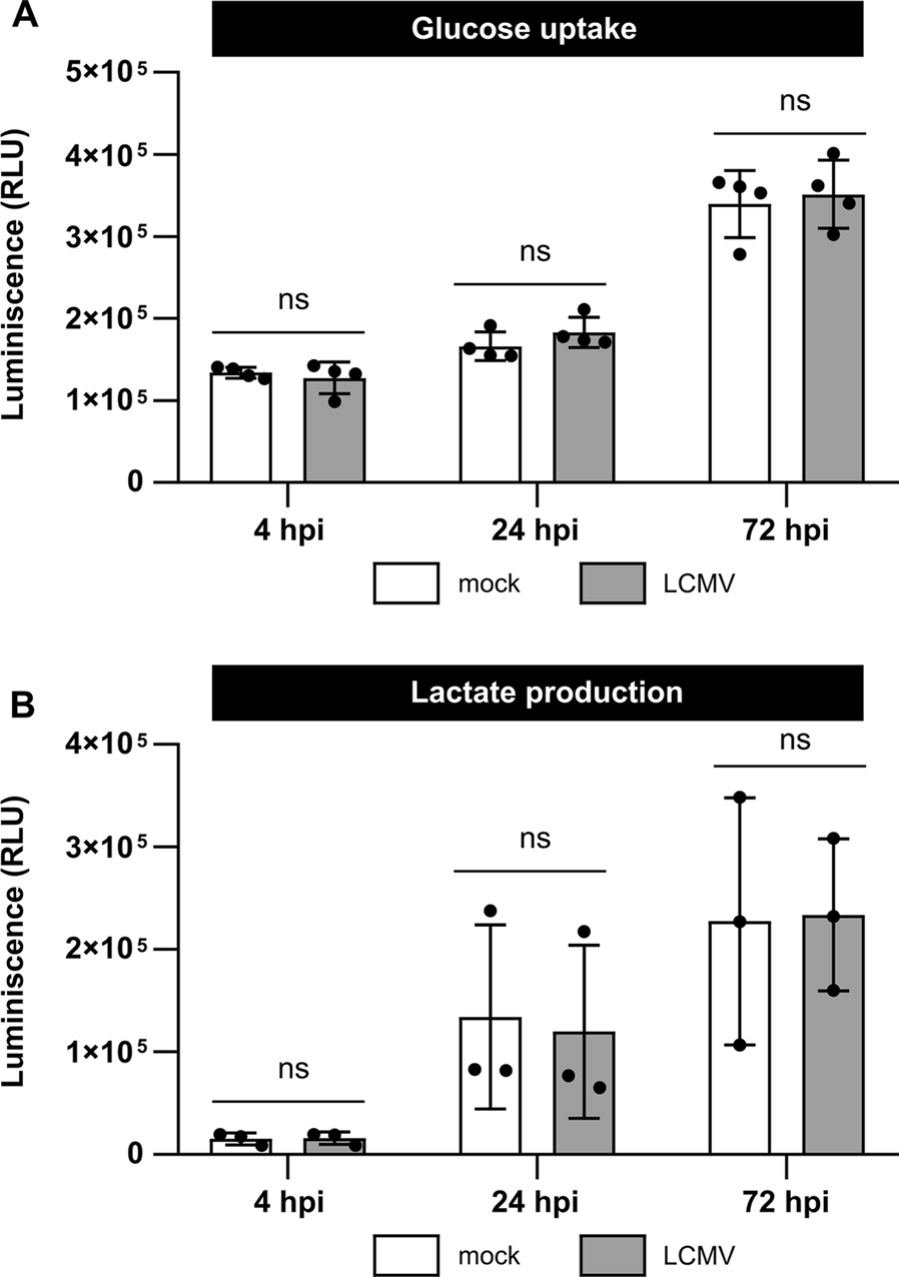
Neither glucose consumption nor extracellular lactate production is affected during LCMV infection. **A** MRC-5 cells were mock- or LCMV-infected at an MOI of 3. Cell lysates were harvested at indicated time after infection, and glucose uptake was measured using the Glucose Uptake-Glo™ Assay. **B** MRC-5 cells were infected as described above, cell culture medium was collected at the indicated time after infection, and the relative lactate amount was measured using the Lactate-Glo™ Assay. Data from three to four independent experiments are shown as a scatter of individual values. Mean and SD are shown as column and error bars, respectively. Statistical differences between groups were analyzed using one-way ANOVA with Sidak’s post hoc test. ns, not significant; hpi, hours post-infection; RLU, relative light unit

Since elevated expression of GLUTs and glycolytic enzymes suggested that LCMV might activate glycolysis, we further examined glucose consumption and lactate excretion following LCMV infection. We hypothesized that LCMV infection increases glucose import but decreases the amount of lactate efflux from the cells. However, as shown in Fig. 3, LCMV-infected cells did not exhibit any effect on glucose uptake (Fig. 3A) or lactate production (Fig. 3B) compared to the mock. Taken together, these findings indicate that LCMV-induced changes in the expression of genes involved in glucose transport and glycolysis do not lead to a detectable increase in glucose uptake and its utilization.

### Exogenous glucose is not required for optimal LCMV propagation

**Fig. 4:**
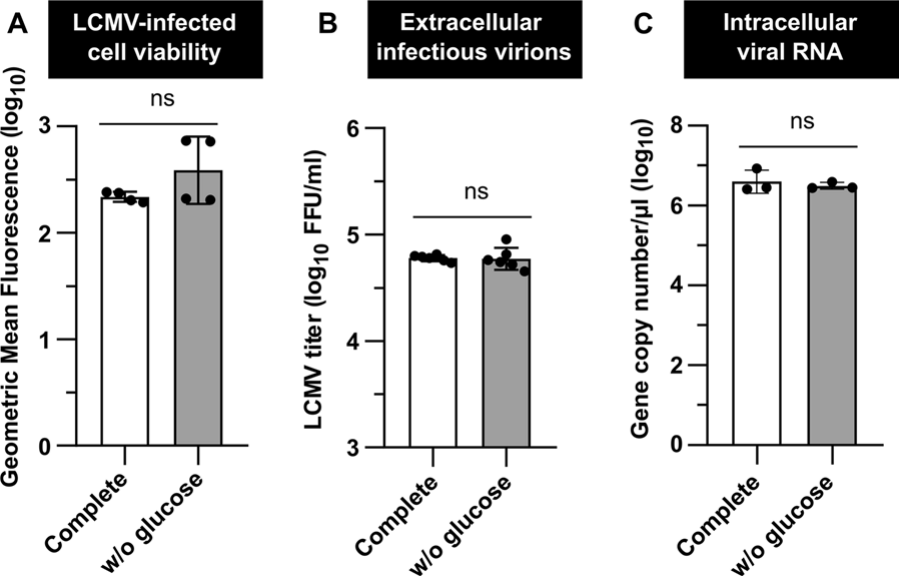
Glucose starvation is not detrimental to LCMV propagation. MRC-5 cells were LCMV-infected at an MOI of 3. After adsorption, the inoculum was removed and replaced with either complete or glucose-free medium (w/o glucose), followed by a 24-h incubation. **A** Twenty-four hours after medium exchange, cell viability was measured by LIVE/DEAD™ Fixable Dead Cell Stain Kit. Data from four independent experiments are shown as a scatter of individual values of geometric mean fluorescence intensity. Mean and SD are shown as column and error bars, respectively. **B** LCMV titer in the extracellular media was determined by a focus-forming assay. Data from six independent experiments are shown as a scatter of individual values. Mean and SD are shown as column and error bars, respectively. **C** Total RNA was isolated, and intracellular LCMV RNA was detected by amplification of the NP gene fragment using RT-qPCR. Copy numbers of viral NP were calculated using a standard curve. Data from three independent experiments are shown as a scatter of individual values. Mean and SD are shown as column and error bars, respectively. Statistical differences between groups were analyzed using unpaired t-test (**C**) or unpaired t-test with Welch’s correction (**A**, **B**). FFU/ml, focus-forming units per milliliter

Even though the glucose uptake is not enhanced during LCMV infection, we aimed to determine if exogenous glucose is an essential carbon source for efficient LCMV propagation. First, we evaluated the survival of LCMV-infected MRC-5 cells under glucose deprivation conditions and found that glucose is dispensable for their optimal cell viability (Fig. 4A). MRC-5 cells were then infected with LCMV at MOI of 3 and subsequently cultivated in either complete medium or medium lacking glucose. Twenty-four hours after medium exchange, the release of extracellular infectious virions and intracellular viral RNA levels were quantified by focus forming assay and RT-qPCR, respectively. As shown in Fig. 4B, C, neither LCMV infectious virion production nor viral replication was significantly reduced in the glucose-free growth medium. These findings demonstrate that glucose, as an extracellular carbon source, is not required for LCMV replication and spread.

### 2-DG inhibits the production of infectious LCMV virions

**Fig. 5:**
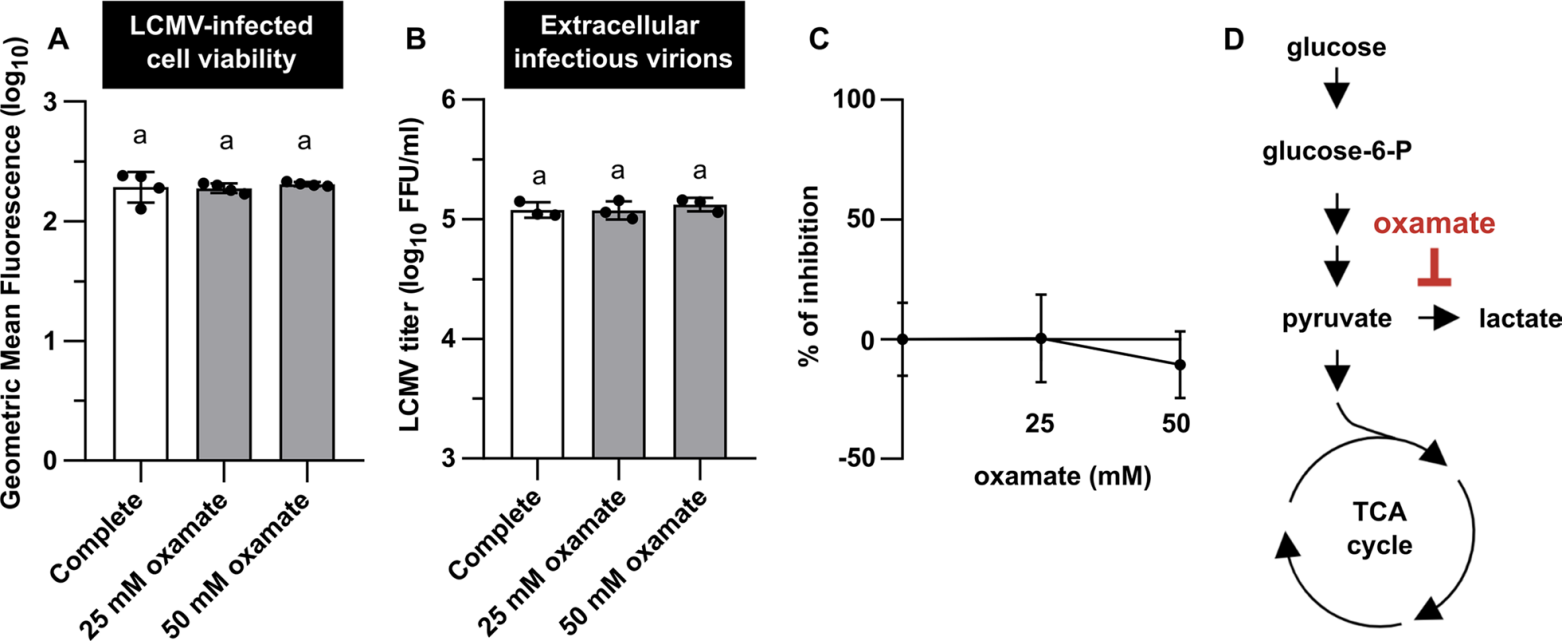
Oxamate does not affect LCMV infectious virion production. MRC-5 cells were infected with LCMV at an MOI of 3. After adsorption, the inoculum was removed and replaced with complete medium or complete medium supplemented with oxamate (25 or 50 mM). **A** Twenty-four hours after medium exchange, cell viability was measured by LIVE/DEAD™ Fixable Dead Cell Stain Kit. Data from four independent experiments are shown as a scatter of individual values. Mean and SD are shown as column and error bars, respectively. **B** LCMV titers in the extracellular media were determined by a focus-forming assay. Data from three independent experiments are shown as a scatter of individual values. Mean and SD are shown as column and error bars, respectively. Statistical differences between groups were analyzed using Welch’s ANOVA with Tamhane’s T2 post hoc test (**A**) or one-way ANOVA and Tukey’s post hoc test (**B**). Different letters above the columns indicate significant differences (*P* ≤  < 0.05), while identical letters indicate no significant difference. **C** The relative (%) inhibitory effect of oxamate on LCMV infection was calculated from LCMV titers shown in **B** (for more details, see Materials and methods). Data are shown as a mean and SD. **D** Simplified schematic representation of glycolysis and tricarboxylic acid cycle with highlighted target of oxamate. FFU/ml, focus-forming units per milliliter

**Fig. 6:**
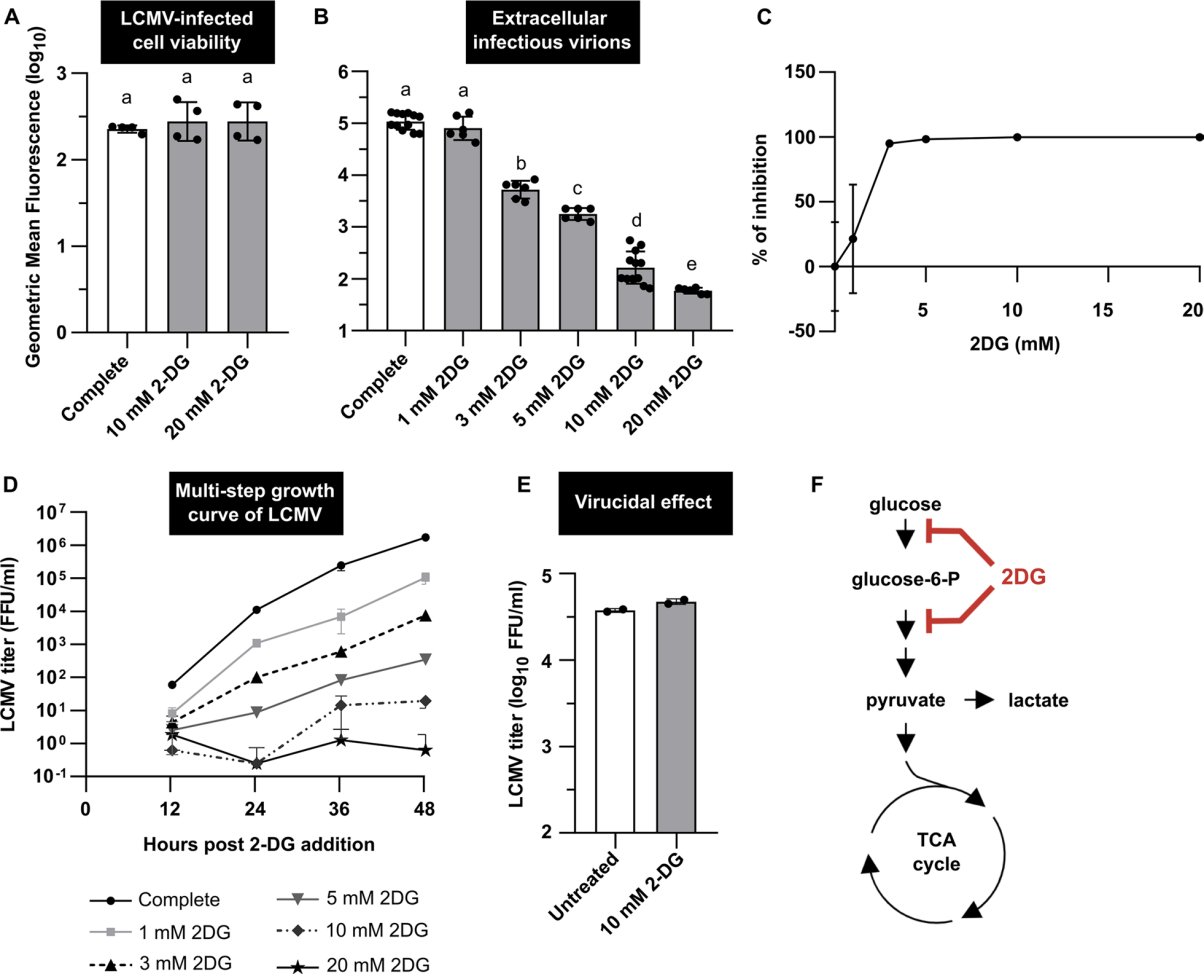
2-DG reduces LCMV infectious virion production in a dose-dependent manner. MRC-5 cells were infected with LCMV at an MOI of 3 (**A**–**C**) or MOI of 0.01 (**D**). After adsorption, the inoculum was removed and replaced with complete medium or complete medium supplemented with 2-DG (1, 3, 5, 10 or 20 mM). **A** Twenty-four hours after medium exchange, cell viability was measured by LIVE/DEAD™ Fixable Dead Cell Stain Kit. Data from four independent experiments are shown as a scatter of individual values. Mean and SD are shown as column and error bars, respectively. **B** LCMV titers in the extracellular media were determined by a focus-forming assay. Data from six to twelve independent experiments are shown as a scatter of individual values. Mean and SD are shown as column and error bars, respectively. Statistical differences between groups were analyzed using Welch’s ANOVA with Tamhane’s T2 post hoc test **(A**, **B**). Different letters above the columns indicate significant differences (*P* ≤ 0.05), while identical letters indicate no significant difference. **C** The relative (%) inhibitory effect of 2-DG on LCMV infection was calculated from LCMV titers shown in **B** (for more details, see Materials and methods). Data are shown as a mean and SD. **D** The multi-step growth kinetics of LCMV was determined from the viral titer analysis in the extracellular media at indicated times by a focus-forming assay. Data from two independent experiments are shown as a mean and SD. **E** Virucidal effect of 2-DG against LCMV (for more details, see Materials and methods). Data from two independent experiments are shown as a scatter of individual values. Mean and SD are shown as column and error bars, respectively. **F** Simplified schematic representation of glycolysis and tricarboxylic acid (TCA) cycle with highlighted targets of 2-DG. FFU/ml, focus-forming units per milliliter

To further investigate the role of glucose metabolism in optimal viral replication, we examined infectious virion production in the presence of 2-DG or oxamate. 2-DG is a glucose analog that inhibits hexokinase and phosphoglucoisomerase involved in the first steps of glycolysis (Fig. 6F), while oxamate is a pyruvate analog inhibiting lactate dehydrogenase (LDH) (Fig. 5D). Importantly, neither 2-DG nor oxamate treatment negatively impacted LCMV-infected MRC-5 viability (Figs. 5A, 6A). Treatment of LCMV-infected cells (single-step infection) with increasing concentrations of oxamate for 24 h had no significant impact on the viral propagation (Fig. 5B, C). Since oxamate competitively inhibits LDH enzymatic activity, thus impairing lactate formation from pyruvate, this result is consistent with our previous findings regarding lactate production (Fig. 3B) and *LDHA* expression (Fig. 2F).

In contrast to oxamate, 2-DG strongly inhibited LCMV infectious virion production during single-step infection of MRC-5 cells in a dose-dependent manner (Fig. 6B, C). We observed a nearly 2.5-log_10_ drop in the release of extracellular infectious virions in the presence of 10 mM 2-DG and a 3-log_10_ decrease in the case of 20 mM 2-DG (Fig. 6B). Additionally, we used these absolute values of viral titers (from Fig. 6B) to calculate the relative inhibition of LCMV infection in the presence of 2-DG. The concentrations of 2-DG at or above 5 mM were found to effectively reduce the production of LCMV virions, with 98.4%, 99.8%, and 99.9% inhibition of viral infection at concentrations of 5, 10, and 20 mM, respectively (Fig. 6C). Surprisingly, similarly as in the case of glucose deprivation, RT-qPCR analysis revealed that 2-DG had no negative effect on intracellular viral RNA copies (Fig. 8D).

The multi-step kinetics of LCMV (MOI 0.01) in the presence of various 2-DG concentrations confirmed the same trends as the single-step infections (Fig. 6D). While the LCMV titer gradually increased in the control conditions (complete medium) up to 48 h, we observed an even more pronounced dose-dependent inhibitory effect of 2-DG than in the case of a single-step infection. Importantly, the presence of 10 or 20 mM 2-DG did not exert any deleterious effect on the viability of LCMV-infected MRC-5 cells during the 48-h treatment (Additional file 2: Fig. S1).

To determine whether the observed antiviral properties of 2-DG could be due to a direct viral inactivation before virion adsorption on the cells, viral particles were pre-incubated with the drug, followed by the analysis of LCMV titer. As shown in Fig. 6E, no virucidal effect of 2-DG was observed. Thus, the negative effect of 2-DG on LCMV is not caused by its direct virucidal properties but rather by its interference with cellular processes affecting viral life cycle.

**Fig. 7:**
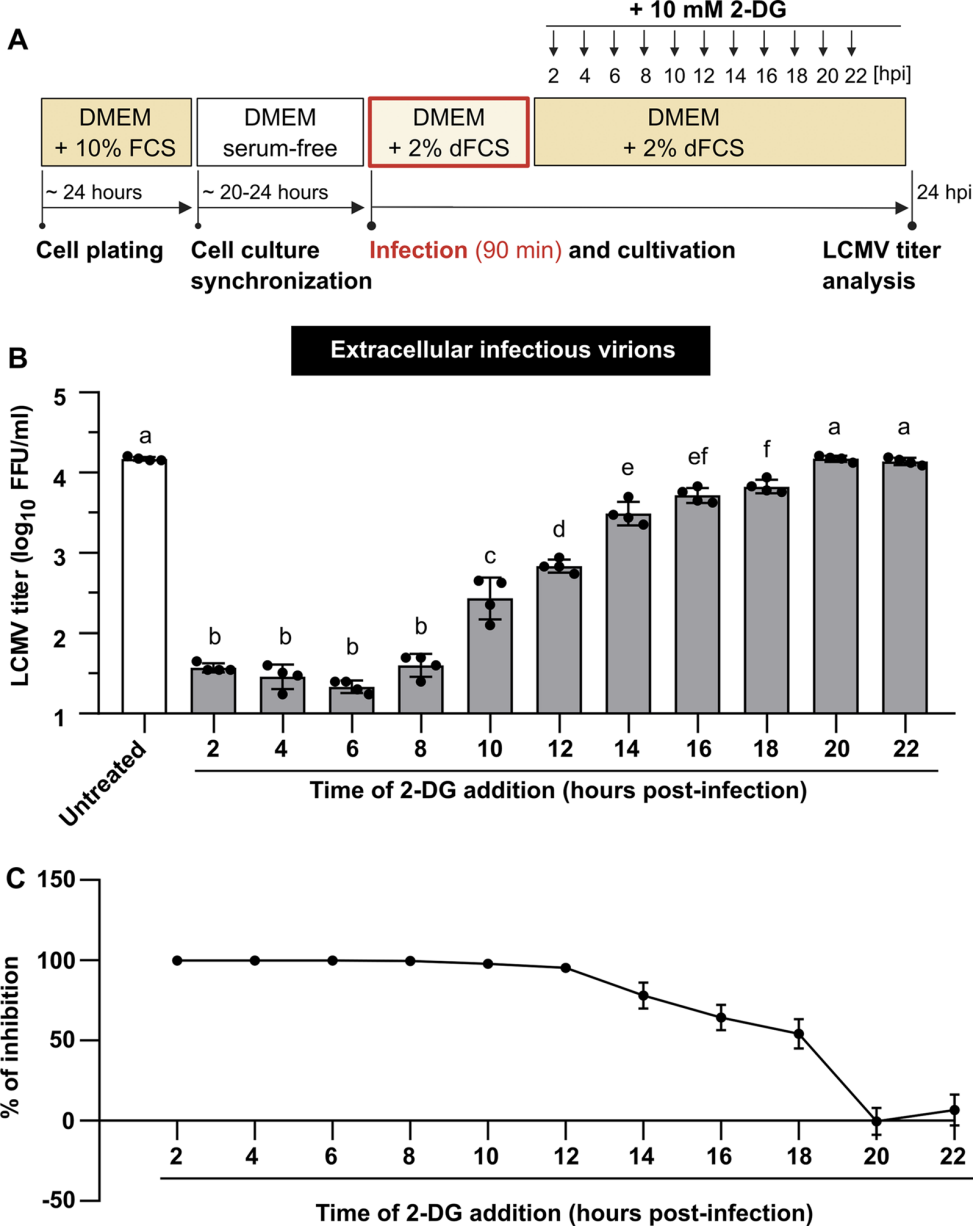
Inhibitory effect of 2-DG on LCMV propagation is dependent on the time of its addition. **A** Schematic of the experimental design. **B** MRC-5 cells were infected with LCMV at an MOI of 3. After adsorption, the inoculum was removed and replaced with complete medium, to which 10 mM 2-DG was added at different time points after infection. Supernatants were collected 24 h after medium exchange and LCMV titers were determined by a focus-forming assay. Data from four independent experiments are shown as a scatter of individual values. Mean and SD are shown as column and error bars, respectively. Statistical differences between groups were analyzed using one-way ANOVA and Tukey’s post hoc test. Different letters above the columns indicate significant differences (*P* ≤ 0.05), while identical letters indicate no significant difference. **C** The relative (%) inhibitory effect of 2-DG on LCMV infection was calculated from LCMV titers shown in **B** (for more details, see Materials and methods). Data from four independent experiments are shown as a mean and SD. FFU/ml, focus-forming units per milliliter

To explore the kinetics of the negative effect of 2-DG on the LCMV life cycle, a time-of-addition study was conducted. MRC-5 cells were infected with LCMV and re-fed with a complete medium to which 10 mM 2-DG was added at various time points post-infection. Samples from all infectious cell-free media were collected after 24 h of cultivation and analyzed by FFA. As shown in Fig. 7B, C, 2-DG had the most significant impact on LCMV propagation when added to the culture up to 8 hpi, with an almost 100% inhibition of viral infection. Regarding the LCMV growth kinetics (Fig. 1), this time range corresponds to the stage prior to virion budding, when viral proteins biosynthesis and virion assembly occur. The addition of 2-DG within 10–18 hpi also caused a significant decrease in the virion production, but the overall viral yield was higher compared to the post-infection period for up to 8 h. A detailed comparison of LCMV titers from the 2-DG time-of-addition study (Fig. 7B, C) and LCMV growth kinetics (Fig. 1) showed that the quantity of infectious virions formed when 2-DG was added in the range of 10–18 hpi might represent approximately the amount that was produced before the treatment with 2-DG started (within the time range up to 10–18 hpi).

Taken together, we showed that 2-DG, but not oxamate, significantly reduces LCMV infectious virion production. Despite a profound inhibitory effect of 2-DG on LCMV titer, it showed no impact on the intracellular viral RNA levels. These observations, together with results from the 2-DG time-of-addition experiment, indicate that a critical point in the LCMV life cycle affected by 2-DG is the assembly and/or budding of infectious virions.

### 2-DG impairs N-glycosylation of viral glycoprotein precursor

**Fig. 8:**
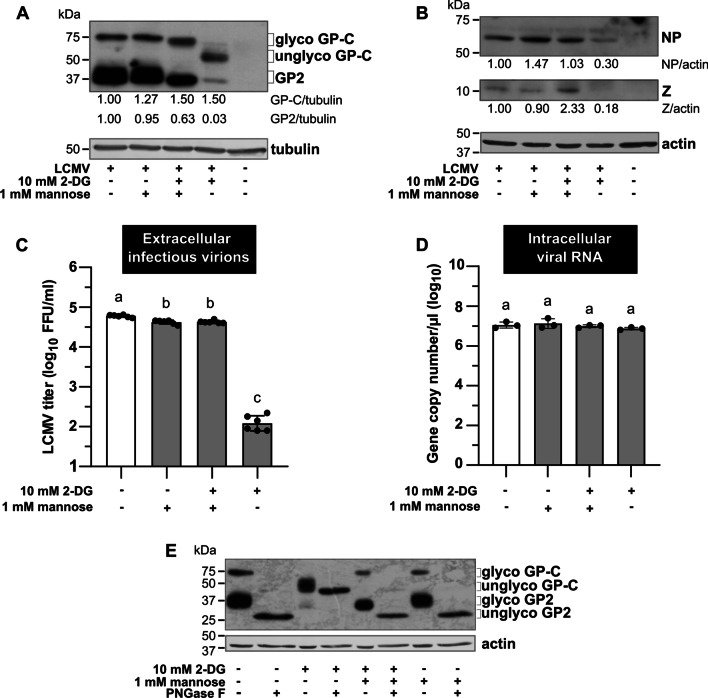
Addition of mannose can reverse the negative impact of 2-DG on the LCMV life cycle. MRC-5 cells were infected with LCMV at an MOI of 3. After adsorption, the inoculum was removed and replaced with fresh media according to experimental design, followed by 24-h incubation. **A**, **B** Whole-cell extracts were harvested and used for the immunoblot analysis of viral protein expression levels using anti-GP2 (**A**), anti-NP, and anti-Z antibodies (**B**). The signals obtained with anti-α-tubulin or anti-β-actin antibodies were used as loading controls. The ratios of GP-C/tubulin, GP2/tubulin, NP/actin, and Z/actin were determined based on the densitometric analysis of protein abundance. Final values are expressed relative to control, which was set to 1. **C** LCMV titers in the supernatants were analyzed by FFA. Data from six independent experiments are shown as a scatter of individual values. Mean and SD are shown as column and error bars, respectively. **D** Total cellular RNA was isolated and LCMV RNA was detected by RT-qPCR. Copy numbers of viral NP were calculated using a standard curve. Data from three independent experiments are shown as a scatter of individual values. Mean and SD are shown as column and error bars, respectively. Statistical differences between groups were analyzed using Welch’s ANOVA with Tamhane’s T2 post hoc test (**C**) or one-way ANOVA with Tukey’s post hoc test (**D**). Different letters above the columns indicate significant differences (*P* ≤ 0.05), while identical letters indicate no significant difference. **E** Whole-cell extracts were subjected to either mock or PNGase F treatment and used for the immunoblot analysis of viral glycoprotein levels using anti-GP2 antibody. The signal obtained with anti-β-actin antibody was used as a loading control. NP, nucleoprotein; Z, Z protein; glyco/unglyco GP-C, glycosylated/unglycosylated glycoprotein precursor complex; GP2, glycoprotein 2; glyco/unglyco GP2, glycosylated/unglycosylated glycoprotein 2; FFU/ml, focus-forming units per milliliter

Given that the absence of exogenous glucose in the medium did not affect LCMV propagation, it seemed unlikely that the observed reduction in infectious virion production in the presence of 2-DG was due to glycolytic flux inhibition. Aside from its effect on glucose metabolism, 2-DG has been shown to interfere with protein N-glycosylation [22]. To understand the mechanism of antiviral effect of 2-DG, we examined the viral GP-C synthesis and processing by western blot analysis. In the lysates from LCMV-infected cells, we observed two differentially migrating bands on the SDS-PAGE gel: slower and faster, corresponding to glycosylated forms of GP-C and GP2, respectively (Fig. 8A). However, the treatment with 2-DG resulted in a distinct shift in the GP-C mobility that might represent its unglycosylated form (Fig. 8A).

To test whether the change in the GP-C mobility was caused by impairment of its glycosylation status, protein lysates were further treated with Peptide-N-Glycosidase F (PNGase F), which cleaves all N-linked glycans from the protein backbone. In control samples, digestion with PNGase F shifted the band corresponding to glycosylated GP-C form to a faster migrating faint band representing its deglycosylated counterpart. Supporting our assumptions, no additional change in GP-C migration in the 2-DG-treated samples was observed when PNGase F was used (Fig. 8E).

Moreover, even though the abundance of unglycosylated GP-C in 2-DG-treated cells was almost comparable to its glycosylated counterpart in control samples, we observed a profound reduction in the GP2 protein level (Fig. 8A). Collectively, these data revealed that 2-DG prevents viral GP-C from N-glycosylation and its further cleavage into GP1 and GP2.

In addition, we noticed that the viral NP and Z protein abundances were also decreased in the LCMV-infected cells treated with 2-DG (Fig. 8B). This is consistent with previous findings showing that 2-DG-mediated inhibition of N-linked glycosylation induces endoplasmic reticulum (ER) stress and an unfolded protein response (UPR), resulting in a general decrease in protein synthesis [23, 24].

The inhibitory effect of 2-DG on protein N-glycosylation was previously shown to be mediated by its structural similarity to mannose. 2-DG competes with mannose for incorporation into dolichol-pyrophosphate-linked oligosaccharide chains that serve as precursors for protein N-glycosylation. Adding mannose to the medium can thus reverse the detrimental effect of 2-DG [22]. As shown in Fig. 8A, coincubation of LCMV-infected cells with both 2-DG and mannose resulted in a considerable recovery of the signal corresponding to the glycosylated form of GP-C. The addition of mannose also restored the protein levels of viral NP and Z (Fig. 8B) and production of infectious virions (Fig. 8C). Moreover, the presence of mannose alone or together with 2-DG did not further affect intracellular viral RNA abundance (Fig. 8D).

**Fig. 9:**
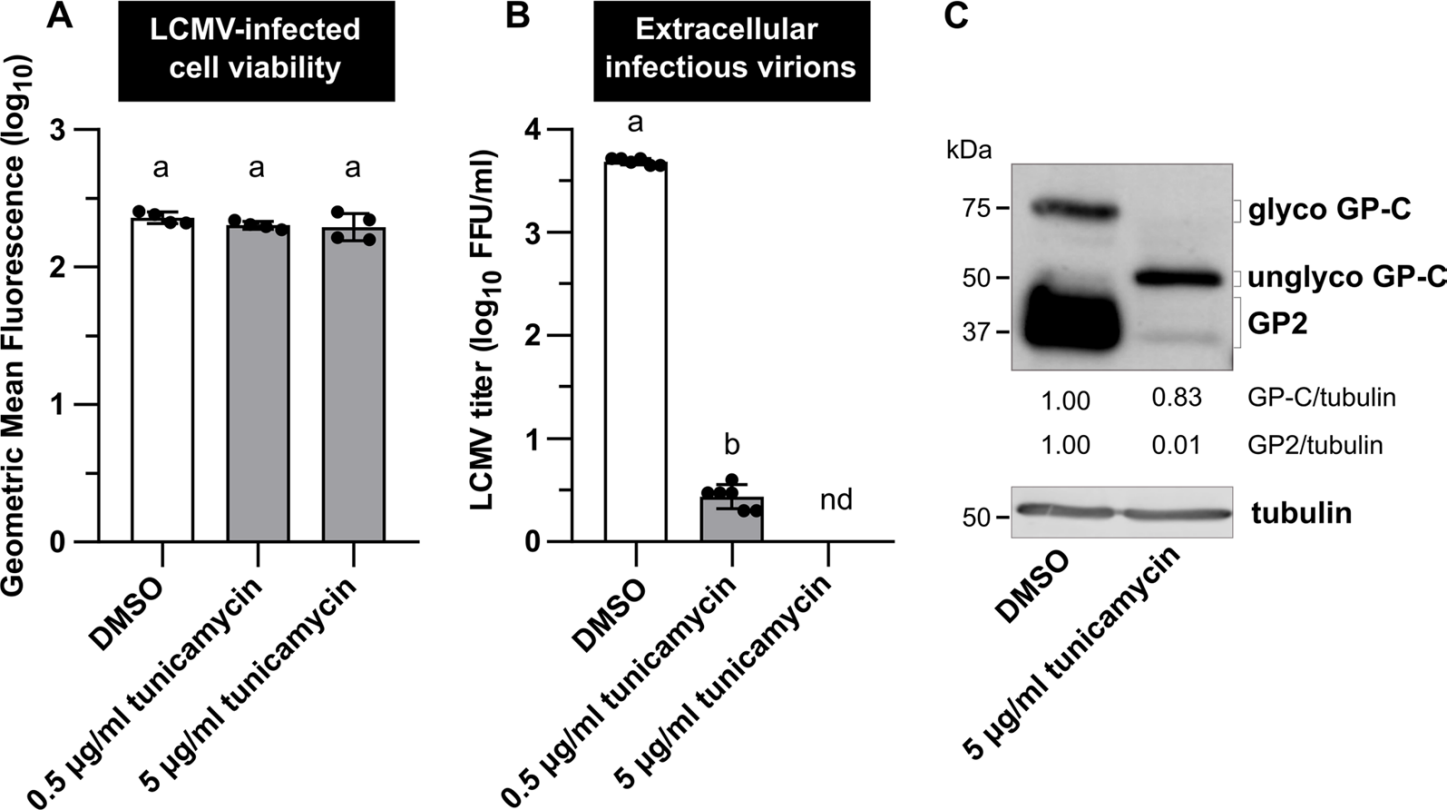
N-glycosylation of the LCMV GP-C is impaired in the presence of tunicamycin. MRC-5 cells were infected with LCMV at an MOI of 3. After adsorption, the inoculum was removed and replaced with complete medium supplemented with 0.5% DMSO or 0.5–5 µg/ml tunicamycin. **A** Twenty-four hours after medium exchange, cell viability was measured by LIVE/DEAD™ Fixable Dead Cell Stain Kit. Data from four independent experiments are shown as a scatter of individual values of geometric mean fluorescence intensity. Mean and SD are shown as column and error bars, respectively. **B** Supernatants were collected 24 h after medium exchange and LCMV titers were determined by a focus-forming assay. Data from six independent experiments are shown as a scatter of individual values. Mean and SD are shown as column and error bars, respectively. Statistical differences between groups were analyzed using one-way ANOVA and Tukey’s post hoc test (**A**) or unpaired t-test with Welch’s correction (**B**). Different letters above the columns indicate significant differences (*P* ≤ 0.05), while identical letters indicate no significant difference. **C** Whole-cell extracts were harvested 24 h after media exchange and used for the immunoblot analysis of viral glycoprotein expression levels using anti-GP2 antibody. The signals obtained with anti-α-tubulin antibody were used as loading controls. The ratios of GP-C/tubulin and GP2/tubulin were determined based on the densitometric analysis of protein abundance. Final values are expressed relative to control (DMSO), which was set to 1. FFU/ml, focus-forming units per milliliter; nd, not detected; GP2, glycoprotein 2; glyco/unglyco GP-C, glycosylated/unglycosylated glycoprotein precursor complex

As a proof of concept that 2-DG targets glycosylation, an experiment using tunicamycin, another N-glycosylation inhibitor known to interfere with LCMV propagation [25], was performed. The effects of tunicamycin on infectious virion production, viral GP-C N-glycosylation, and its further cleavage were similar to those observed with 2-DG (Fig. 9B, C). Importantly, the concentrations of tunicamycin used during the 24-h treatment had no effect on LCMV-infected cell viability (Fig. 9A).

Taken together, our data revealed that 2-DG inhibits LCMV life cycle primarily through interfering with protein N-linked glycosylation, as the addition of mannose efficiently reversed its antiviral effect. The 2-DG treatment further affected viral protein synthesis and processing of GP-C, which presumably impaired the correct assembly and/or budding of infectious virions.

## Discussion

A considerable number of recent studies have reported that a wide variety of viruses modulate cellular metabolism in a manner similar to cancer cells. Inducing the Warburg effect-like phenotype is one of the most common metabolic alterations triggered by viral infection that helps cells to cope with the high demand for energy and macromolecule biosynthesis (e.g., nucleotides, lipids or amino acids) needed for efficient viral propagation [19, 21, 26–30]. We have shown recently that persistent LCMV infection of human cells increased the accumulation of alpha-enolase (a glycolytic enzyme converting 2-phosphoglycerate to phosphoenolpyruvate), activated serine/threonine kinase Akt, and enhanced transactivation of the HIF-1α transcription factor [31]. Both phosphatidylinositol 3-kinase (PI3K)/Akt signaling and HIF transcription factors are known to play a crucial role in the upstream regulation of glycolysis [32], while alpha-enolase is a typical HIF-1α target gene [33].

Though previous studies have identified metabolic changes triggered by many families of RNA viruses, *Arenaviridae*-mediated rewiring of central carbon metabolism has not yet been elucidated. Here we aimed to investigate alterations of glucose metabolism during distinct stages of acute LCMV infection. We found that expression of the glucose transporters GLUT1 and GLUT3, as well as of the first rate-limiting glycolytic enzymes HK1 and HK2, is upregulated in LCMV-infected cells at 24 hpi, which corresponds to the peak of infectious virion production (Figs. 1, 2A–D). Several viruses have been shown to induce the expression and/or activity of glucose transporters and hexokinases. For instance, rhinovirus infection rapidly increases GLUT1 expression and glucose uptake in the infected cells in a PI3K-dependent manner [20]. Increased expression of GLUT1 and HK2 was also observed during Dengue virus infection [21]. Similarly, Kaposi's sarcoma-associated herpesvirus (KSHV) infection induces GLUT3 and HK2 expression [19]. Human immunodeficiency virus type 1 (HIV-1) increases the expression of not only GLUT1 and GLUT3 but also GLUT4 and GLUT6 in CD4+ T cells. Upregulation of glucose transporters induces enhanced glucose uptake and hexokinase activity, which coincides with the upregulation of HK1, but not HK2, in HIV-1-infected cells [34]. Interestingly, GLUT1, GLUT3, and GLUT4, together with HK2 expression, were also significantly enhanced during severe acute respiratory syndrome coronavirus 2 (SARS-CoV-2) infection of Vero E6 cells [35].

As the increased expression of glucose transporters and hexokinases in LCMV-infected cells may reflect enhanced glycolytic flow, we sought to examine if glucose carbons preferentially enter the TCA cycle or are converted to lactate and metabolized in a manner of aerobic glycolysis. At 24 hpi, we observed a slight increase in *PDHA1* mRNA levels in response to LCMV infection, while *LDHA* transcription was suppressed (Fig. 2E, F). These findings indicate that glucose carbons might be preferentially processed by PDH, allowing them to enter the TCA cycle. In line with this hypothesis, we found that LCMV infection had no impact on lactate excretion (Fig. 3B).

All identified changes in the expression of glucose transporters and enzymes involved in glucose metabolism suggested a virus-induced increase in glucose consumption. Surprisingly, we observed that LCMV infection did not affect glucose uptake (Fig. 3A). Furthermore, depriving LCMV-infected cells of glucose supply by its depletion from the culture medium had no significant impact on virus yield (Fig. 4B). Similarly, oxamate, an LDH inhibitor, had no effect on infectious virus production (Fig. 5B, C), which was consistent with our findings on *LDHA* expression (Fig. 2F) and lactate production in LCMV-infected cells (Fig. 3B). This was unexpected as most viruses that modify the expression of metabolic enzymes involved in glycolysis also exhibit an increased need for the supply and metabolization of glucose [19–21, 34]. The findings presented here, on the other hand, suggest that glucose is not a critical carbon source for LCMV replication and that the observed changes in the expression of key glycolytic enzymes and transporters are most likely a non-specific cellular response to infection.

2-DG is primarily used as a glucose analog, which is converted by hexokinase to 2-DG-6-phosphate (2-DG-6-P) that cannot be further metabolized via glycolysis. Inhibition of glycolysis by 2-DG thus mimics glucose deficiency; therefore, both of these conditions usually have a similar effect on viral replication [20, 21]. It is well known that 2-DG and glucose deprivation trigger ER stress and metabolic stress (mainly caused by ATP depletion), thus negatively affecting cancer cell growth and survival [23, 36, 37]. However, neither glucose-free conditions nor 2-DG treatment had a negative impact on the LCMV-infected MRC-5 cell viability under tested conditions (Figs. 4A, 6A). There are at least two possible explanations for these results: (1) MRC-5 cells are non-cancerous fibroblasts lacking Warburg-like metabolic reprogramming typically observed in tumor cells, which makes them less susceptible to related metabolic stresses [38, 39]; (2) the cell doubling time for MRC-5 line is approximately 36–72 h [40] that is significantly longer than the treatment durations used in our study. Therefore, a putative effect of treatment on their growth or viability might not be detectable within short time periods.

Although depriving LCMV-infected cells of exogenous glucose did not affect infectious virion production, 2-DG treatment significantly reduced viral yield (Fig. 6B–D). Based on these findings, it was evident that LCMV propagation is dependent on another intracellular process that is impaired by 2-DG, independently of glycolysis inhibition. In cells, 2-DG is converted not only to 2-DG-6-P but also to GDP-2-DG and UDP-2-DG. As a result, 2-DG, in the form of GDP-2-DG, is an effective competitor of GDP-mannose, a required substrate to assemble lipid-linked oligosaccharides on the ER membrane needed for protein N-glycosylation [41]. Consistent with this hypothesis, our data shows that 2-DG's inhibitory effect on LCMV propagation primarily interferes with N-linked glycosylation of viral glycoprotein rather than reducing glycolysis. Indeed, the addition of mannose completely reversed the detrimental effect of 2-DG on LCMV infectious virion production (Fig. 8C), GP-C glycosylation and cleavage (Fig. 8A, E) and protein abundance of viral NP and Z (Fig. 8B). Reversal of the inhibitory effect of 2-DG on N-linked glycosylation of viral glycoproteins by mannose has also been demonstrated in other enveloped viruses, such as Semliki Forest virus [42], herpes simplex virus 1 [43], influenza virus A [41], KSHV [24] or SARS-CoV-2 [35].

**Fig. 10:**
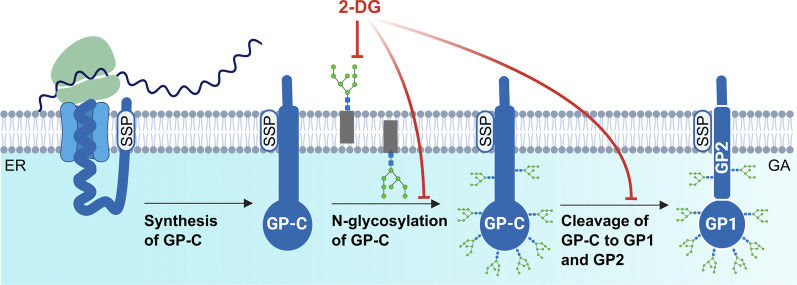
Schematic of the 2-DG-mediated impairment of viral GP-C N-glycosylation and cleavage. LCMV glycoprotein is synthesized as a single glycoprotein precursor complex (GP-C) with N-terminal stable signal peptide (SSP) that targets the protein to the endoplasmic reticulum (ER) and is subsequently co-translationally cleaved. Post-translationally, GP-C undergoes extensive N-glycosylation and cleavage into GP1 and GP2, which occurs in the Golgi apparatus (GA) or post-Golgi compartment. The mature tripartite complex SSP/GP1/GP2 forms a functional spike glycoprotein of LCMV. 2-deoxy-D-glucose (2-DG) competes with mannose for incorporation into lipid-linked oligosaccharide precursors that are assembled at the membrane of the ER and further used for protein N-glycosylation. As a result, in the presence of 2-DG, synthesized GP-C is not glycosylated, which has a negative impact on its cleavage into GP1/GP2 and the formation of functional spike glycoproteins. Created with BioRender.com

Viral surface glycoproteins of enveloped viruses play an important role, especially in the initial stages of viral infection, mediating the interaction with host cell receptors and the subsequent entry of viruses into the intracellular environment [44]. A deficiency in glycosylation of viral glycoproteins may have varying consequences on the course of viral infection, depending on their structure and maturation process. Aberrant N-glycosylation of viral glycoprotein can interfere with its cleavage, transport, folding, or overall production of functional virions [25, 42, 45, 46]. Wright et al. found that treating LCMV-infected cells with tunicamycin inhibits glycosylation of viral GP-C, prevents its cleavage, and limits the production of infectious virions. According to electron microscopy images, the presence of tunicamycin prevented the complete budding of virions [25]. Since 2-DG reduced N-linked glycosylation and cleavage of GP-C in the same manner as tunicamycin (Fig. 9C), we can assume that infectious LCMV virion production is also disrupted in the presence of 2-DG. Another explanation for reduced viral yield could be that virions are produced and shed but are non-infectious due to incorrect GP-C processing. Such an effect has been reported in the presence of tunicamycin during Junín virus propagation, another member of the *Arenaviridae* family [46]. Our data show that 2-DG treatment also reduces viral NP and Z protein levels, implying that decreased titers are caused rather by lowered virion production than defective virions. Moreover, the effect of 2-DG on LCMV multiplication was the most detrimental when added to the cultivation medium within the first 8 h post-infection (Fig. 7B, C). Adding 2-DG at later post-infection periods allowed us to detect a certain viral yield (Fig. 7B), which was, however, comparable to the number of virions produced in a time range before the addition of inhibitor, as shown by comparison to viral growth kinetics in Fig. 1. Thus, we conclude that in LCMV-infected cells, 2-DG treatment predominantly affects viral GP-C N-glycosylation and processing, without affecting glucose metabolism, which subsequently impairs viral assembly and budding (Fig. 10).

Interestingly, we detected two counterintuitive observations in our data. Firstly, the overall level of viral RNA was not affected during either 2-DG treatment or glucose deprivation (Figs. 4C, 8D). Numerous studies have shown that a drop in viral yield caused by nutrient starvation or the presence of 2-DG is usually positively correlated with a decreased amount of viral RNA in the infected cells [21, 47–49]. On the contrary, our results suggest that within the 24-h period, 2-DG interferes with LCMV infectious virion release from the cells but does not affect viral replication. Secondly, the expression level of GP-C was not reduced in the 2-DG-treated LCMV-infected cells, unlike NP and Z protein levels (Fig. 8A, B). 2-DG is known to function as UPR and ER stress inducer (see below), which leads to general translational arrest in the cells, which may explain the drop in NP and Z protein abundances. However, GP-C of LCMV was shown to induce UPR regardless of 2-DG, ensuring optimal viral multiplication [50]. It is not clear why GP-C synthesis is unaffected by translational arrest; therefore, it would be interesting to speculate if there is some selective avoidance of GP-C transcripts from the general translational block. In summary, the unexpected results described above indicate a more complex molecular story behind the 2-DG-mediated anti-LCMV effect, which will require further study.

Inhibition of protein N-glycosylation by 2-DG or tunicamycin generally results in an accumulation of non-glycosylated and misfolded proteins in the ER, thereby inducing ER stress and triggering the UPR pathway [23]. UPR activation is a frequent feature of viral infection, caused mostly by enhanced protein synthesis in the ER or even by the use of ER membranes to form viral replication complexes [51, 52]. Some viruses manipulate the host UPR and selectively activate or inhibit only some of its signaling branches in order to establish optimal conditions for replication [50, 53, 54]. However, global activation of UPR may also have a general antiviral effect, as demonstrated in the case of 2-DG-induced UPR during KSHV or SARS-CoV-2 infection [24, 35]. Acute, but not persistent, LCMV infection selectively activates the ATF6-regulated branch of UPR but avoids induction of PERK and IRE1 branches. Moreover, extensive production of viral GP-C in the ER was shown to be responsible for the observed activation [50]. Unlike LCMV, 2-DG acts non-selectively and activates all UPR branches [22, 24]. Thus, the inhibitory effect of 2-DG on the production of infectious LCMV virions may be mediated not only by insufficient glycosylation of the viral GP-C but also by the overall activation of UPR. Further research is necessary to assess the precise mechanism of 2-DG action during LCMV infection.

Taken together, the 2-DG-mediated inhibition of LCMV propagation observed in our experiments broadens the prospects for arenavirus antiviral therapy. The negative effect of aberrant N-glycosylation of GP-C on LCMV virion production has only been shown in studies with tunicamycin, which is unsuitable for therapeutic purposes due to its high toxicity [55]. In contrast, no toxicity of 2-DG was detected in the cell culture at the concentrations used in our study. The safety of 2-DG on healthy cells relies on various factors, such as the concentration and duration of exposure, as well as the metabolic state of the cell. Although 2-DG blocks glycolysis, suppresses protein glycosylation, and induces cellular stress responses that can affect cellular metabolism, biosynthesis, and signaling, low concentrations and short-term exposure typically do not pose significant risks to normal cells [56].

As a result, 2-DG has been studied in over 200 clinical trials for the treatment of various tumors, and it has been shown to be safe and well-tolerated by patients [57, 58]. 2-DG is unique as an antiviral drug since it inhibits glycolysis and interferes with N-linked glycosylation during viral infection. It can reduce viral infections in both enveloped and non-enveloped viruses. Therefore, it is not surprising that in the midst of the recent global health crisis, a 2-DG oral administration has acquired emergency approval in India to help control SARS-CoV-2 infection. Clinical trial results demonstrated that 2-DG helps in the faster recovery of hospitalized patients and reduces their dependence on supplementary oxygen [59]. Based on its mechanisms of action, safety record in humans, and availability, 2-DG appears to be suitable for further investigation and development for clinical application in infection by arenaviruses and other emerging viruses.

The main limitation of the presented study is the exclusive use of the in vitro cell-culture model. Even though in vitro research has many advantages and allows us to perform experiments under tightly controlled chemical and physical conditions, we are aware that this is far beyond the complexity of biological systems, such as the human body. Therefore, our study may serve as a basis for future research in the field of arenavirus therapy using in vivo models.

## Conclusions

In summary, for the first time, we conducted a study on the interaction between arenaviral infection (specifically LCMV, as a representative member) and host cell glucose metabolism. Despite not finding any dependence of the LCMV life cycle on glucose supply or utilization, we reaffirmed the importance of protein N-glycosylation for productive LCMV infection. 2-DG potently reduces LCMV propagation by inhibiting N-linked protein glycosylation, regardless of inhibiting glycolysis, highlighting the potential for a new targeted antiviral therapy that might be applicable for a broader spectrum of arenaviruses.

## Supplementary Information

**Additional file 1: Table S1.** List of used primers

**Additional file 2: Figure S1.** Viability of the LCMV-infected MRC-5 cells cultivated in the presence of 2-DG for 48-hours.

## Data Availability

All the data generated during this study was statistically analyzed and is included in this published article. Raw datasets and materials described in the article are available from the corresponding author upon reasonable request.
