# Supplementary material for: 2-Deoxy-D-glucose inhibits lymphocytic choriomeningitis virus propagation by targeting glycoprotein N-glycosylation
Source: Virol J. 2023 May 31;20:108. doi: 10.1186/s12985-023-02082-3 (PMC10231856; doi:10.1186/s12985-023-02082-3)
Supplement: Supplementary file 1 — Additional file 1: Table S1. List of used primers [file 12985_2023_2082_MOESM1_ESM.docx]

**Table S1** List of primers

| **Gene** | **Forward primer (5’ – 3’)** | **Reverse primer (5’ – 3’)** |
| --- | --- | --- |
| *ACTB* (β-actin) | TCCTCCCTGGAGAAGAGCTA | ACATCTGCTGGAAGGTGGAC |
| *GLUT1* | CTCCTTTCTCCAGCCAGCAATG | CCAGCAGAACGGGTGGCCATA |
| *GLUT3* | CTGGAGAGGTTAAGGTGCAATAA | TCCCTGGACTCCATCCAAAATTA |
| *HK1* | AATGCTGGGAAACAAAGGT | AGAGGAATCCCTTCTTGGT |
| *HK2* | GAGCCACCACTCACCCTACT | CCAGGCATTCGGCAATGT |
| *PDHA1* | TTCTCAGAAGCCGGCAAGC | ACTCCATTCGGCGTACAGTC |
| *LDHA* | TGGCAGCCTTTTCCTTAGAA | ACTTGCAGTTCGGGCTGTAT |
| *NP* (LCMV) | GATCAAAAACAATTCAAGCAAGATT | GTCCCACACTTTGTCTTCATACTCC |
