# Supplementary material for: 2-Deoxy-D-glucose inhibits lymphocytic choriomeningitis virus propagation by targeting glycoprotein N-glycosylation
Source: Virol J. 2023 May 31;20:108. doi: 10.1186/s12985-023-02082-3 (PMC10231856; doi:10.1186/s12985-023-02082-3)
Supplement: Supplementary file 2 — Additional file 2: Figure S1. Viability of the LCMV-infected MRC-5 cells cultivated in the presence of 2-DG for 48-hours. [file 12985_2023_2082_MOESM2_ESM.docx]

**
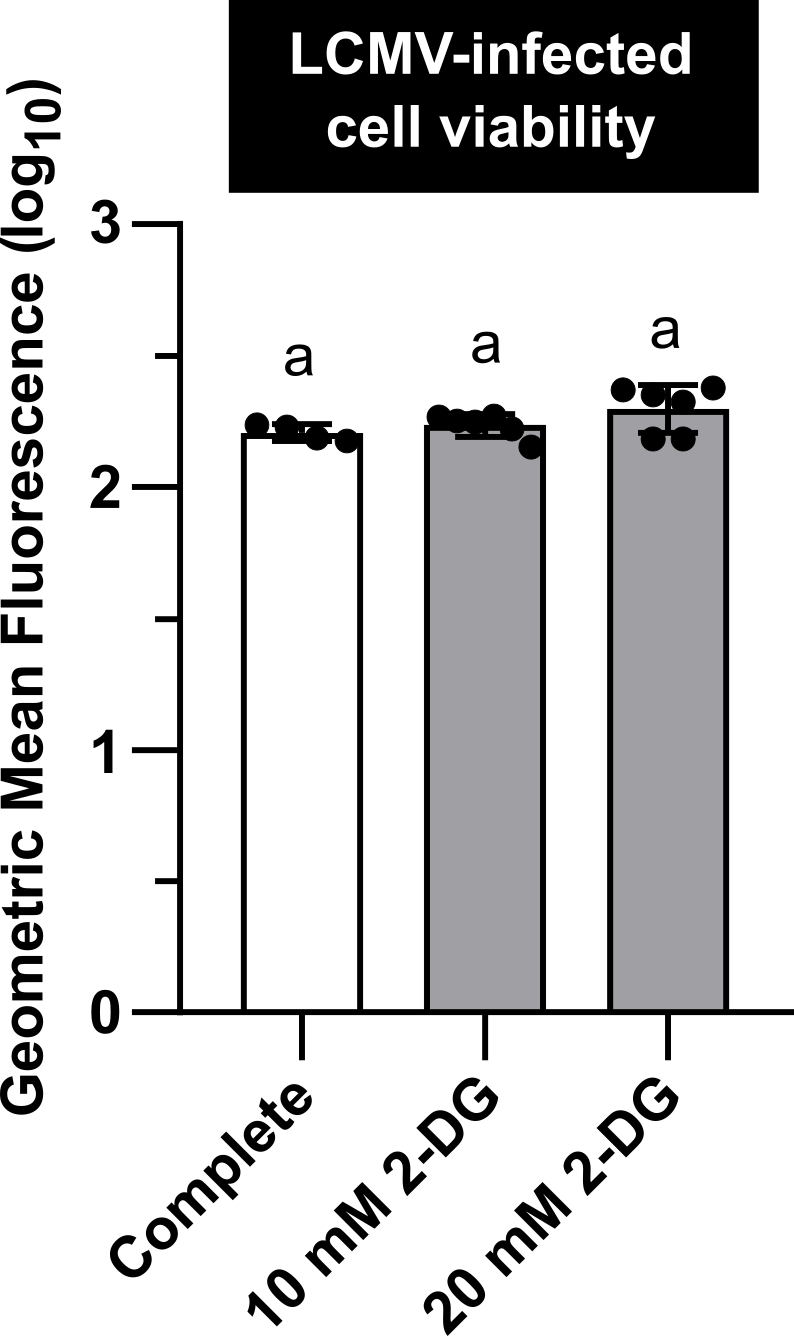
**

**Fig S1. Viability of the LCMV-infected MRC-5 cells cultivated in the presence of 2-DG for 48-hours.** MRC-5 cells were LCMV-infected at an MOI of 0.01. After adsorption, the inoculum was removed and replaced with fresh media according to experimental design. Forty-eight hours after medium exchange, cell viability was measured by LIVE/DEAD^TM^ Fixable Dead Cell Stain Kit. Data from four to six independent experiments are shown as a scatter of individual values of geometric mean fluorescence intensity. Mean and SD are shown as column and error bars, respectively. Statistical differences between groups were analyzed using Welch’s ANOVA with Tamhane’s T2 post hoc test. The same letter above columns indicate there is no significant difference between them.
